# Select Congenital Heart Disease: Important Echocardiographic Features and Changes during Pregnancy

**DOI:** 10.31083/j.rcm2403066

**Published:** 2023-02-22

**Authors:** David S. Majdalany, Francois Marcotte

**Affiliations:** ^1^Department of Cardiovascular Diseases, Mayo Clinic, Scottsdale, AZ 85259, USA

**Keywords:** echocardiopgraphy, pregnancy, congenital heart disease

## Abstract

Congenital heart disease (CHD), which affects 1% to 2% of all births, is the most common abnormality in women contemplating pregnancy in western countries. With diagnostic and interventional advances, most patients with CHD survive into adulthood and require lifelong cardiac follow-up with cardiac imaging, particularly echocardiography and cardiac computed tomography. Multiple hemodynamic and physiologic changes of pregnancy may predispose patients with CHD to clinical decompensation and an inability to tolerate pregnancy. This manuscript reviews common CHD lesions, their repair or palliative interventions, long-term sequelae, important features to assess on cardiac imaging, and the impact of pregnancy on these types of lesions. Moreover, the review bridges the fields of CHD, cardiac imaging, and maternal cardiology, which will aid clinicians in counseling patients and managing pregnancies.

## 1. Introduction

Congenital heart disease (CHD) affects about 1% of the population and varies in complexity from simple valvular anomalies and septal defects to complex defects associated with severe hemodynamic disturbances that may be incompatible with life. With advances in early diagnosis and therapeutic procedures, most children with CHD would survive into adulthood. Moreover, adults with CHD now substantially outnumber children, and in the Western world, CHD is the most common cardiovascular disease complicating pregnancy. In this review, we cover select CHD lesions, palliative interventions with their inherent long-term sequelae and need for follow-up, important cardiac imaging findings (predominantly echocardiography), and the impact and outcomes of pregnancies of patients with these abnormalities.

**Fig. 1.**
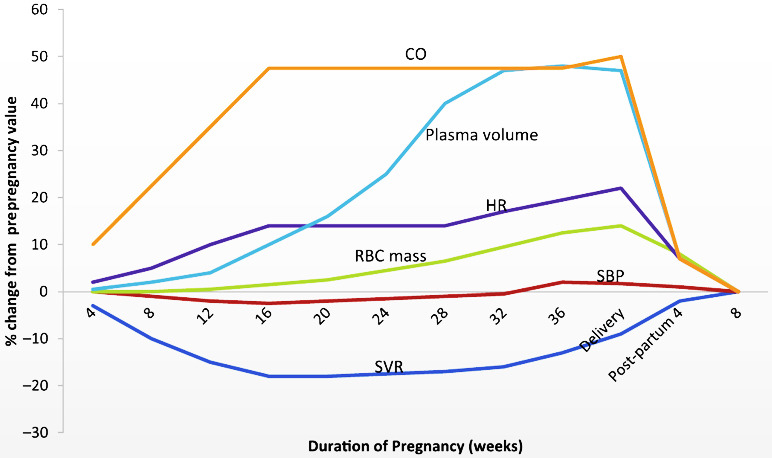
**Normal hemodynamic and physiologic changes of pregnancy**. (From Bhatt and DeFaria Yeh [1]; used with permission.) During pregnancy, plasma volume, red blood cell mass, cardiac output, and heart rate increase, whereas systemic vascular resistance and blood pressure decrease. CO, cardiac output; HR, heart rate; RBC, red blood cell; SBP, systolic blood pressure, SVR, systemic vascular resistance.

During pregnancy, several adaptative mechanisms (including increased cardiac output and blood volume) occur that foster fetal and placental development but place substantial stress on the maternal heart and circulation (Fig. 1, Ref. [1]), which may precipitate clinical decompensation. An increase in plasma volume during pregnancy is accompanied by increases in erythropoietin and red cell mass. Moreover, systemic vascular resistance (SVR) decreases, and cardiac output increases to 30% to 50% above baseline, peaking in the latter part of the second trimester and further increasing at delivery. The increase in cardiac output is related to increased stroke volume from increased plasma volume and heart rate, and at time of delivery to further heart rate increases from contractions and associated pain and autotransfusion. Postpartum, SVR rapidly increases, which may precipitate heart failure in patients with compromised SVR.

Proper clinical assessment before conception and during pregnancy can substantially benefit from noninvasive imaging that brings important diagnostic and prognostic information for risk stratification. Of the various noninvasive imaging techniques available, transthoracic echocardiography (TTE) is radiation free and the safest with the highest temporal resolution (30–60 frames/second). TTE provides accurate assessment of dynamic cardiac anatomy and function as well as hemodynamic changes associated with the pregnant state. In this review, we will describe our TTE methodology for assessing the pregnant patient with either suspected or established native or repaired select CHD, focusing on the most common lesions, including intracardiac and extracardiac shunt lesions, left heart obstructive lesions, right heart lesions, and transposition complexes. We will also describe TTE’s limitations and guide the clinician as to when to request further investigation, for example using 3-dimensional (3D) techniques such as cardiac magnetic resonance imaging (CMR). Noninvasive techniques devoid of ionizing radiation, such as TTE and CMR, are safe throughout pregnancy, although the latter exposes mother and fetus to a strong external magnetic field and is generally performed after embryogenesis is complete (12 weeks) and always performed without gadolinium contrast. CMR possesses a larger field of view and does not have bone (rib) or air (lung) interference as does ultrasonography and has better spatial resolution (1–2 mm) than TTE (2 mm), although the temporal resolution is lower (20–30 frames/second). Cardiac computed tomography (CCT) has the highest (<1 mm) spatial resolution but the lowest temporal resolution (10–15 frames/second) and uses ionizing radiation that is best avoided during pregnancy, especially during the first trimester unless providing lifesaving imaging information.

## 2. Normal Echocardiographic Findings in Pregnancy

Physiologic changes of pregnancy can be evaluated by TTE (Table 1, Ref. [2]). As venous return and cardiac output increase, biatrial dimension, atrioventricular valve annular diameter, and biventricular end-diastolic dimension and volume increase, with a corresponding increase in ventricular mass, ventricular outflow tracts, and great vessel size [3]. Although left ventricular (LV) ejection fraction is unchanged during pregnancy, strain imaging suggests that contractility increases in the first and second trimesters but then decreases, reaching a nadir at 36 weeks continuing to 6 weeks postpartum. The LV filling pressures remain normal based on measurements of mitral annular early diastolic velocity, but with a decreased E wave and E wave/A wave ratio [4]. The LV stroke volume and cardiac output increase as do transvalvular velocities and gradients, with estimated pulmonary artery (PA) systolic pressure remaining in the physiologic range [5].

**Table 1.** **Physiologic changes on echocardiography during pregnancy**.

Unchanged in pregnancy	Increased in pregnancy	Normal in pregnancy
Ejection fraction	LVEDD	Pericardial effusion (often trace to mild)
Fractional shortening	LV mass	Pseudodyskinesis
Peak myocardial systolic velocity	Cardiac output	
Average systolic SR	RV diastolic area	
E/E′ ratio	LA volume	
RVSP	LA size	
	RA size	
	Valve annulus dimension	
	Aortic and pulmonary VTI	

Abbreviations: E/E′ ratio, ratio of early diastolic transmitral flow velocity to tissue Doppler mitral annular early diastolic velocity; LA, left atrium; LV, left ventricle; LVEDD, left ventricular end-diastolic dimension; RA, right atrium; RV, right ventricle; RVSP, right ventricular systolic pressure; SR, strain rate; VTI, velocity time integral (From Afari *et al*. [2]; used with permission.)

## 3. Intracardiac and Extracardiac Shunts

Successfully repaired or native cardiac shunts not associated with pulmonary hypertension, such as atrial septal defect (ASD), partial anomalous pulmonary venous return (PAPVR), restrictive ventricular septal defect (VSD), or patent ductus arteriosus (PDA) are generally well-tolerated throughout pregnancy, although some studies suggest increased neonatal and maternal events [6, 7]. When there are hemodynamically significant pre-tricuspid shunts, for example ASD and PAPVR with pulmonary to systemic flow ratio (Qp:Qs) ≥2, volume overload will occur in the right-sided chambers, as shown on 2-dimensional (2D) imaging by right atrial and right ventricular (RV) dilatation. Usually biventricular systolic function is preserved, although diastolic interventricular septal flattening is commonly present, which reflects increased diastolic tricuspid flow. For posttricuspid shunts, the driving pressure for a left to right shunt is systemic, either ventricular in the case of VSD (characterized by systolic shunting) or arterial in the case of PDA or aortopulmonary window (characterized by continuous systolic-diastolic shunting). In neonates, the shunt initially causes left-sided volume overload, which can manifest as heart failure if the shunt is hemodynamically significant. If uncorrected, such shunts lead to progressive pulmonary vascular injury, microvascular obstruction, and pulmonary hypertension that will become irreversible, usually within 2 years, and cause right-sided pressure overload (Eisenmenger syndrome), associated with high (>50%) maternal mortality and are thus a contraindication to pregnancy [8].

### 3.1 Atrial Septal Defect

**Fig. 2.**
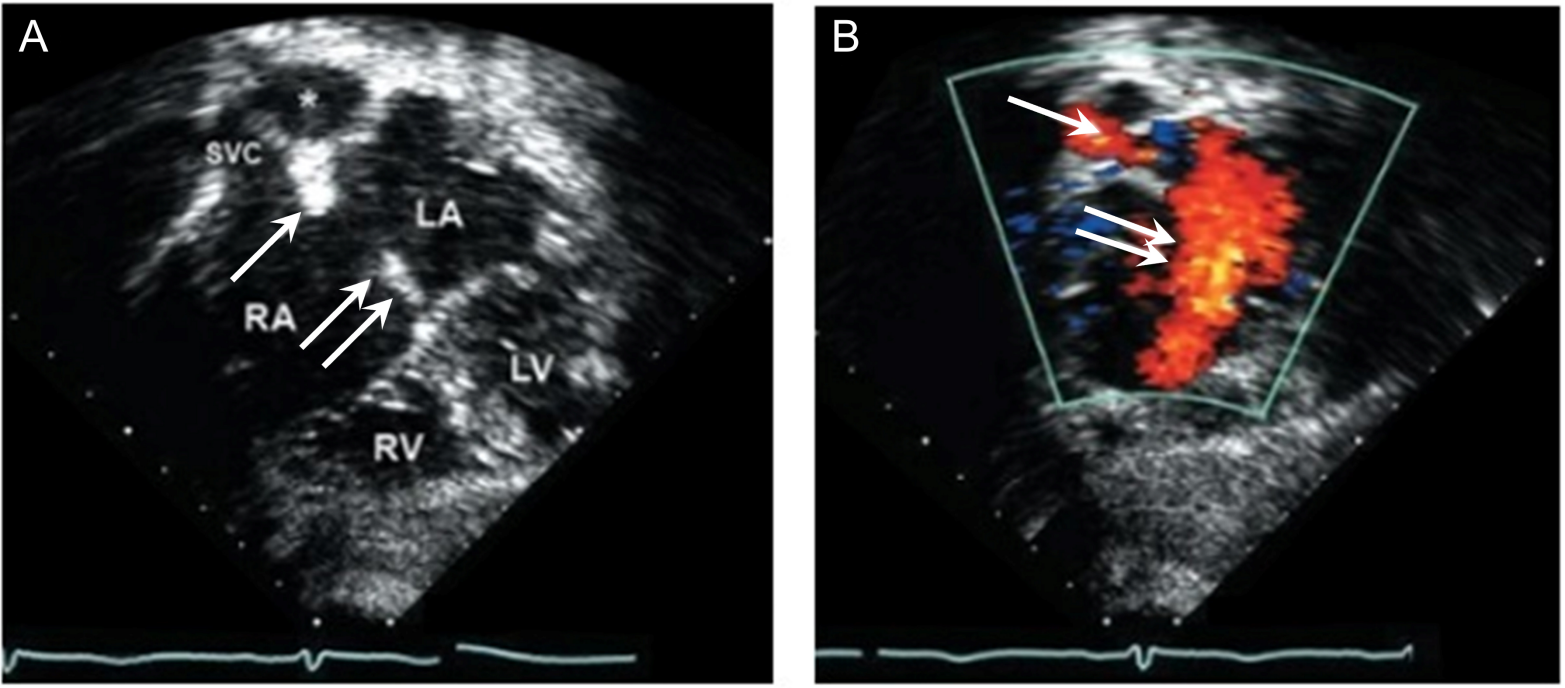
**Subcostal imaging of a secundum ASD**. (Modified from Cabalka AK. Abnormalities of atria and atrial septation. In [10]; used with permission of Mayo Foundation for Medical Education and Research.) (A) Echocardiography in the subcostal coronal plane showing drop-out in the interatrial septum consistent with a moderately sized secundum ASD. In this plane, the rims of the defect appear well developed (single arrow, superior edge; double arrow, inferiorly). The right PA (asterisk) can be seen just posterior to the SVC. (B) Left to right shunting can be seen on color flow Doppler through the ASD (double arrow) and the right pulmonary vein to the LA (single arrow). ASD, atrial septal defect; LA, left atrium; LV, left ventricle; RA, right atrium; RV, right ventricle; SVC, superior vena cava.

Guidelines for interatrial shunts have been developed to differentiate ASD from atrioventricular septal defect (AVSD) and patent foramen ovale [9]. For ASD, the most specific window for shunt assessment remains biplane interrogation in the subcostal window, which places the interatrial septum perpendicular to the ultrasound beam in both the 4-axis and short-axis views and can show the presence of a septal solution of continuity, motion abnormalities (aneurysm defined as >10 mm excursion), or a prior patch or occluder device. A key echocardiographic finding would be drop-out in the atrial septum, which should be confirmed by using an additional imaging plane (Fig. 2, Ref. [10]). The most specific color flow sign of an interatrial shunt is continuous flow on both sides of the septum, confirmed by pulsed-wave Doppler showing a triphasic left to right flow with late systolic, mid-diastolic, and atrial peaks. Comprehensive scanning of the interatrial septum inferosuperior in the 4-chamber view and left to right in the short-axis view to the bicaval view enables identification of over 95% of hemodynamically significant ASDs. The most sensitive and specific way to diagnose interatrial shunting echocardiographically remains by using agitated saline contrast injected into a peripheral vein.

Secondary functional tricuspid regurgitation can be seen on color-flow Doppler because of the dilated tricuspid annulus and consequent RV and right atrial remodeling. Increased pulmonary valvular flow is normally present in high-output states, such as pregnancy, but may be disproportionately high compared with aortic flow when substantial intracardiac left to right shunting is present. If present, coexistent pulmonary valve stenosis (PS) may further increase pulmonary flow velocities. A PAPVR is another pretricuspid systemic to pulmonary shunt, which can occur either alone or in association with ASD. PAPVR is challenging to diagnose by TTE because pulmonary veins are the most posterior intracardiac structures and are surrounded by lung tissue (air) and situated far from the chest wall (bone) and TTE probe. Diagnosis requires optimal subcostal and suprasternal windows and a high index of suspicion; it may be noted as continuous color flow entering the distal superior or inferior caval vein in the short-axis, subcostal bicaval view. The suprasternal coronal (called *crab*) view of the left atrium is challenging to obtain but may confirm normal centripetal pulmonary venous return to the left atrium. Anomalous left PAPVR to the innominate bridge through a vertical vein would present as cephalad, continuous red-orange flow (toward the probe) lateral to the aortic arch, whereas anomalous right PAPVR to the superior vena cava (SVC) would present as blue flow (away from the probe). If PAPVR is strongly suspected, but not conclusively shown by TTE, cinematic or static (spin echo and noncontrast-enhanced gated angiography) CMR can provide better diagnostic imaging of the posteriorly situated pulmonary veins as well as flow quantitation by velocity mapping to calculate (Qp:Qs).

### 3.2 Atrioventricular Septal Defect 

Most adult patients with AVSD have a partial or restrictive AVSD characterized either by a nonrestrictive interatrial shunt (also known as primum ASD) or by a restrictive or repaired interventricular shunt (also called inlet VSD). Women with nonrestrictive interventricular shunts complicated by pulmonary hypertension are generally discouraged from becoming pregnant. Patients with partial or repaired AVSD who become pregnant have more general maternal (23%) and obstetric (21%) complications, more often bear children who are small for gestational age (10%) and have increased risk for recurrence of CHD (10%) [7]. Partial AVSD with an interatrial shunt is generally physiologically comparable to that of ASD (RV volume overload), although anomalies may occur, such as cleft mitral valve, mitral regurgitation (MR), and “gooseneck” left ventricular outflow tract (LVOT) deformity, which is occasionally associated with subaortic stenosis (see below) and accessory mitral valve attachments to the interventricular septum [9]. Although in theory severe MR may be well tolerated with decreased LV afterload associated with pregnancy, substantial prepartum MR appears to increase peripartum interventions, and for many women, MR may worsen postpartum [7]. CMR is rarely needed in AVSD but may be useful to quantitate Qp:Qs or MR severity by velocity mapping [11].

### 3.3 Ventricular Septal Defect

**Fig. 3.**
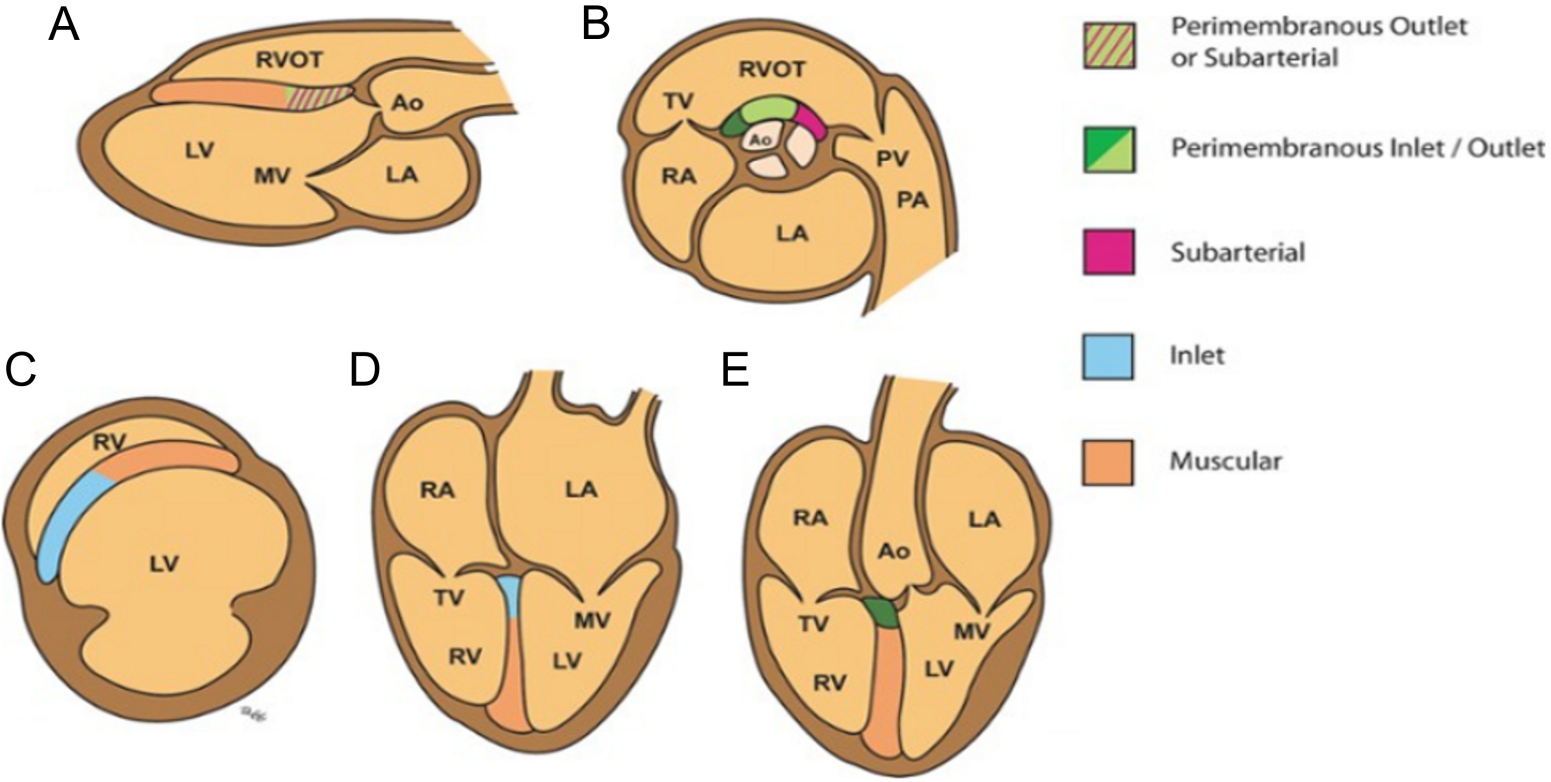
**Echocardiographic assessment of various VSDs**. (From Gelehrter S, Thorsson T, and Ensing G. Ventricular septal defects. In [13]; used with permission of Mayo Foundation for Medical Education and Research.) (A) Parasternal long-axis view showing muscular, perimembranous outlet, and sub-arterial VSDs. (B) Parasternal short-axis view at the base showing perimembranous and sub-arterial VSDs. (C) Parasternal short-axis view at the level of the LV papillary muscles showing inlet and muscular VSDs. (D) Apical 4-chamber view showing inlet and muscular VSDs. (E) Apical 5-chamber view showing muscular and perimembranous VSDs. Ao, aorta; LA, left atrium; LV, left ventricle; MV, mitral valve; PA, pulmonary artery; PV, pulmonary valve; RA, right atrium; RV, right ventricle; RVOT, right ventricular outflow tract; TV, tricuspid valve; VSD, ventricular septal defect.

Most adult patients with VSD have either a restrictive or repaired defect [12]. Fig. 3 (Ref. [13]) shows the various types of VSD that can be seen on standard echocardiographic imaging planes. A solution of continuity can be difficult to identify on 2D echocardiography, which makes color flow Doppler important for the diagnosis of VSD because it can reveal mosaic turbulent flow, thus confirming patch dehiscence or a restrictive defect [14]. Most VSDs are of the perimembranous (75%), outlet (5%), or inlet types (5%, also called partial AVSD), and they can be seen in the basal parasternal short-axis view and confirmed in the orthogonal long-axis or apical 4-chamber and 3-chamber views. Muscular VSD can be seen by comprehensive color flow–guided parasternal short-axis scanning down to the apex and confirmed by orthogonal long-axis parasternal or apical views. Continuous-wave Doppler placed parallel to color flow usually confirms (but may also overestimate) the interventricular gradient. Peak systolic gradients >64 mm Hg generally indicate small restrictive defects, whereas those <25 mm Hg indicate nonrestrictive VSD [15].

Doppler echocardiography (see below) can be used to identify defects associated with VSDs, such as septal aneurysm, LVOT obstruction (LVOTO), RV outflow tract obstruction (RVOTO), aortic stenosis, regurgitation from a bicuspid aortic valve (BAV) or from aortic sclerosis with VSD-caused cusp retraction, and aortic arch obstruction or coarctation. A septal aneurysm typically occurs in the membranous septum, visible as a >7.5 mm rightward bulge in the basal short-axis view. Maternal complications and obstetric events, such as prematurity, can occur in patients with isolated membranous ventricular septal aneurysm (MVSA), although this is unlikely. Generally, the events occur because of other underlying conditions, such as VSD, ventricular dysfunction, pulmonary hypertension, and atrioventricular valve disease (mitral or tricuspid regurgitation) [16]. CMR is rarely needed in VSD but may be useful to quantitate Qp:Qs or severity of valvular regurgitation by velocity mapping.

### 3.4 Patent Ductus Arteriosus 

Most adult patients with PDA have a tiny, inaudible, or repaired defect. However, patients with clinically audible PDAs safely undergo percutaneous closure [15, 17]. PDAs can usually be identified only by color flow imaging and appear as retrograde flow in the main PA on the basal short-axis view or the modified suprasternal long-axis view of the aortic arch, with a left and downward tilt. When flow into the PA is interrogated by continuous-wave Doppler, continuous flow with systolic accentuation will be seen, reflecting the aortopulmonary gradient. A small PDA is well tolerated during pregnancy [15, 17]. Because PDAs are situated deep in the thorax and surrounded by lung, CMR can provide better diagnostic imaging than TTE as well as flow quantitation by velocity mapping to calculate (Qp:Qs).

## 4. Coarctation of the Aorta and Bicuspid Aortic Valve

### 4.1 Coarctation of the Aorta

Coarctation of the aorta occurs in 7% of all cases of CHD and is defined by an aortic arch obstruction (usually proximal descending) with a >20 mm Hg gradient as measured by catheterization and is virtually always associated with hypertension [15, 18]. Coarctation can be assessed clinically by blood pressure measurements in the upper and lower extremities, with a blood pressure gradient ≥20 mm Hg indicating significant obstruction, although the severity may be altered by the presence of collateral vessels.

Adults with coarctation have usually undergone a repair in childhood or infancy, typically by end-to-end anastomosis, whereas those who had repairs as adults typically underwent coarctectomy with an interposition graft. Percutaneous coarctoplasty has been an emerging treatment for both repaired and native coarctation in adolescents and adults [19]. Hypertension occurs more commonly after repaired coarctation, either from a residual obstruction or reduced aortic arch compliance, and also occurs more frequently during pregnancy (5%–30%) [17, 20, 21] as a result of increased cardiac output, particularly if patients have not undergone repair. Echocardiography should be obtained before pregnancy and considered with every trimester follow-up during pregnancy depending on the patient’s clinical status. Women with unoperated coarctation or residual hypertension have a greater risk of major adverse events and hospital admissions, including for uncontrolled hypertension and more often undergo nonurgent cesarean section [21]. Fortunately, most series have not shown repaired coarctation to be associated with increased mortality [17, 22, 23]. The best way to assess the thoracic aorta completely from the aortic valve to the abdominal aorta and diagnose coarctation noninvasively remains by 3D imaging, either by CMR or CCT. During pregnancy, computed tomography and gadolinium contrast are best avoided, but CMR without contrast can be safely performed during pregnancy (typically after the first trimester).

**Fig. 4.**
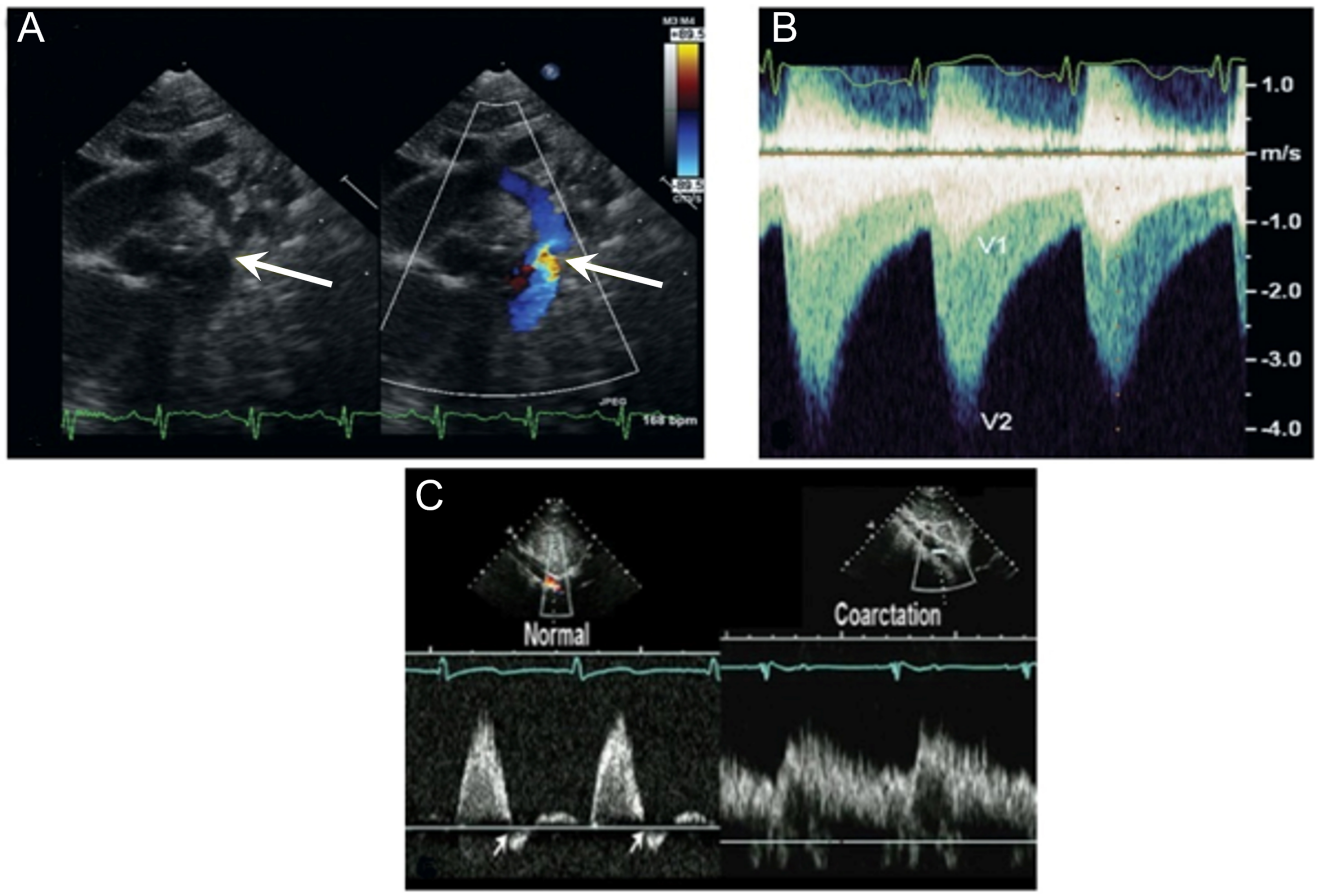
**Echocardiographic evaluation of coarctation of the aorta**. (Modified from Patel A and Young LT. Abnormalities of the aortic arch. In [10]; used with permission of Mayo Foundation for Medical Education and Research.) (A) 2-dimensional imaging (left) shows a discrete coarctation (arrow). Color flow (right) shows aliased flow in the area of obstruction (arrow). (B) Continuous-wave Doppler signal, obtained with a non-imaging probe from the suprasternal notch, shows the characteristic “sawtooth” flow pattern, with antegrade flow extending into diastole. Flow proximal to the coarctation (V1) and high-velocity flow across the coarctation site (V2) can be seen. (C) Pulsed-wave Doppler evaluation of the abdominal aorta at the diaphragm shows a brisk upstroke and downstroke with the presence of an early diastolic flow reversal (EDR) signal (arrows). This signal excludes a diagnosis of proximal obstruction in the thoracic aorta. The right portion shows delayed upstroke and downstroke and absent EDR, indicating coarctation.

By TTE, the suprasternal echocardiographic imaging planes are useful for determining aortic arch geometry (Romanesque, Gothic, or Crenel) and for excluding hypoplasia or discrete stenosis [24]. Color flow Doppler would show flow acceleration and often aliasing artifacts that continue through systole and diastole (Fig. 4A,B, Ref. [10]). The use of guided and nonguided continuous-wave Doppler is often necessary. Peak systolic proximal descending aortic flow velocity >3 m/sec and diastolic flow extension >1 m/sec suggest a hemodynamically significant coarctation, usually associated with abdominal diastolic flow extension. Color flow imaging in the suprasternal plane can reveal collateral aortic flow, typical with substantial coarctation. Note, however, that certain conditions may increase proximal descending aortic flow velocities, including high-output states, significant aortic regurgitation, and, of course, pregnancy. When substantial coarctation is present, additional imaging of the abdominal aorta in the subcostal window reveals a dampened low-velocity signal with flow extension into diastole, with no early diastolic flow reversal (Fig. 4C, Ref. [10]).

In addition to diagnosing hemodynamically significant native coarctation or recoarctation, a comprehensive assessment of the thoracic aorta should be performed at every transthoracic examination starting with the parasternal long-axis view to exclude aortic root and ascending aortic dilatation. By echocardiography, the aorta is typically measured in diastole from leading edge to leading edge, although most other techniques such as computed tomography measure the maximal aortic diameter, typically in systole [25]. Additionally, aortic valve disease or other left-sided heart obstruction should be sought in patients with coarctation and corrected, if moderate or severe before conception, especially if the patient is symptomatic.

Frequently associated left heart obstructive lesions include BAV (>75%), supravalvular aortic stenosis, discrete subaortic stenosis, congenital mitral stenosis (either from a parachute mitral valve or a supravalvular ring or arcade), and cor triatriatum; some of these lesions can be overlooked, and their combination is known as Shone syndrome [26]. Significant obstructive left heart lesions likely increase the incidence of complications in pregnant women. Fortunately, most (>80%) adults with Shone syndrome undergo repair in childhood, but nearly 40% require reoperation and over 30%, a third surgical procedure [27]. Turner syndrome (45,X0) is also associated with coarctation. It is characterized by dysmorphic features like short stature, webbed neck, and wide carrying (forearm) angle. In its pure form, Turner syndrome is associated with infertility, but certain patients may undergo in-vitro fertilization or have a mosaic form of the syndrome and become pregnant. The most serious complication associated with coarctation or Turner syndrome is ascending aortic dissection, which may occur at smaller aortic dimensions in Turner syndrome in patients with small body surface area (BSA) making indexing ascending aortic dimensions to BSA preferable, with >25 mm/$m^{2}$ associated with increased risk of aortic dissection [28]. Marfan syndrome shares with Turner syndrome the increased propensity for aortic dissection but not coarctation or BAV. This connective tissue disorder is characterized by a deficient fibrillin-1 gene and is associated with aortic aneurysms and mitral valve prolapse with aortic and MR. Marfan syndrome is also associated with several extracardiac anomalies described in the Ghent criteria [29] such as ocular lens dislocation, musculoskeletal anomalies (e.g., pectus carinatum or excavatum, joint hypermobility, and dural ectasia). Aortic dilatation >45 mm in patients with Marfan syndrome has been linked to pregnancy-associated aortic dissection and is generally the threshold to perform aortic root and/or ascending aortic graft replacement before conception. In properly selected individuals, the incidence of maternal aortic dissection is low (<2%) but nevertheless associated with a slight increase in aortic size [30]. Although cesarean section is more frequently performed for patients with Marfan syndrome to prevent pushing during delivery, it is associated with greater blood loss. Women with aortic diameters can have safe vaginal deliveries if aortic dimensions are <45 mm [31].

### 4.2 Bicuspid Aortic Valve 

BAV, which frequently occurs with coarctation, is the most common congenital cardiac malformation, occurring in 1% of the general population. Inheritance is autosomal dominant but with variable penetrance, leading more commonly to aortic stenosis (70%) than aortic regurgitation (30%) [32]. The classic appearance of a BAV is a diastolic prolapsing and systolic doming of the aortic valve in the parasternal long-axis view with an ovoid opening in the short-axis view. The most common presentation is bicommissural (>90%) with 3 aortic sinuses and 2-cusp fusion (type 1)—generally the right and left (ten- and four-o’clock closure line), followed by right and noncoronary-cusp fusion (1 and 7-o’clock closure line), and rarely noncoronary and left-cusp fusion (9 and 3-o’clock closure line) [32]. A type 2 BAV (<7%) has 2 aortic sinuses and a 2-cusp aortic valve of equal size and shape. A type 3 BAV has partial fusion of 2 of 3 cusps and 3 aortic sinuses [32]. Color flow imaging shows turbulent diastolic flow in the LVOT from regurgitation (proportional to the long-axis width of the vena contracta jet or short-axis jet area) or supravalvular turbulent systolic flow resulting from stenosis.

Aortic valve stenosis and regurgitation are further suggested by LV remodeling (concentric hypertrophy in significant aortic stenosis and eccentric hypertrophy in significant regurgitation) [33, 34, 35, 36, 37, 38]. Aortic stenosis with a mean gradient >40 mm Hg or an aortic valve area <0.6 ${\mathrm{cm}}^{2}$/$m^{2}$ should be addressed before a woman conceives, as should severe aortic regurgitation with a severely dilated LV (end-diastolic diameter >75 mm or measured volume >80 mL/$m^{2}$ or end-systolic diameter >55 mm or volume >40 mL/$m^{2}$) [18]. Ventricular dysfunction is an ominous and poor prognostic sign for patients with aortic stenosis or regurgitation and should be addressed before pregnancy is contemplated. Aortic root dilatation is frequently associated with BAV and poses an additional risk for further dilation or dissection during pregnancy, especially for patients with a baseline ascending aortic diameter >45 mm. A diameter >50 mm is deemed prohibitive for successful pregnancy and should be repaired before a woman conceives [15, 18]. However, studies of the ascending aorta in high-risk patients, such as those with Marfan and mosaic Turner syndrome, have not shown substantial changes in diameter before and after pregnancy but highlight the risks of dissection in those with clinically significant pregravid aortic dilatation [39, 40].

Women who have undergone mechanical aortic valve replacement sometimes choose to become pregnant, but pregnancy carries substantial maternal and fetal risks [23], including the risk associated with anticoagulation. For these women, a decision must be made to continue warfarin and risk fetal embryopathy or change to a heparin-based regimen in the first trimester or the late third trimester before delivery. Echocardiography is key to assessing prosthetic valve function and dysfunction, and practice standards have been well described [41]. Given the increased physiologic demand of pregnancy on the heart (increased blood volume and cardiac output among other changes), higher prosthetic valve gradients are to be expected and must be differentiated from prosthetic valve dysfunction. Although contrast-enhanced computed tomography is extremely useful in the diagnosis of prosthetic valve dysfunction from pannus or thrombus, it entails substantial radiation exposure that poses a teratogenic risk and is best avoided during pregnancy, especially in the first trimester unless the benefits outweigh the risk to save a pregnant woman’s life. Transesophageal echocardiography can also be useful for diagnosing prosthetic valve dysfunction, especially mitral [41].

Discrete subaortic stenosis can be associated with atrioventricular canal defect and coarctation and is best seen via the parasternal long-axis and apical 3-chamber views, the latter being used for Doppler gradient measurement. Women with a peak gradient >50 mm Hg, a mean gradient >30 mm Hg, or progressive aortic regurgitation should be advised to undergo surgical repair or resection before pregnancy, as gradients will increase substantially during pregnancy [15, 18, 42].

The parasternal short-axis view at the level of the papillary muscle is best for identifying a parachute mitral valve, which will appear as a single papillary muscle and an asymmetric and tilted mitral valve opening. The transmitral gradient, which increases with increased heart rate and cardiac output, can be measured by Doppler using the color-guided, apical 4-chamber view. Women with severe mitral stenosis are best advised against pregnancy [15, 23, 43, 44].

## 5. Tetralogy of Fallot 

**Fig. 5.**
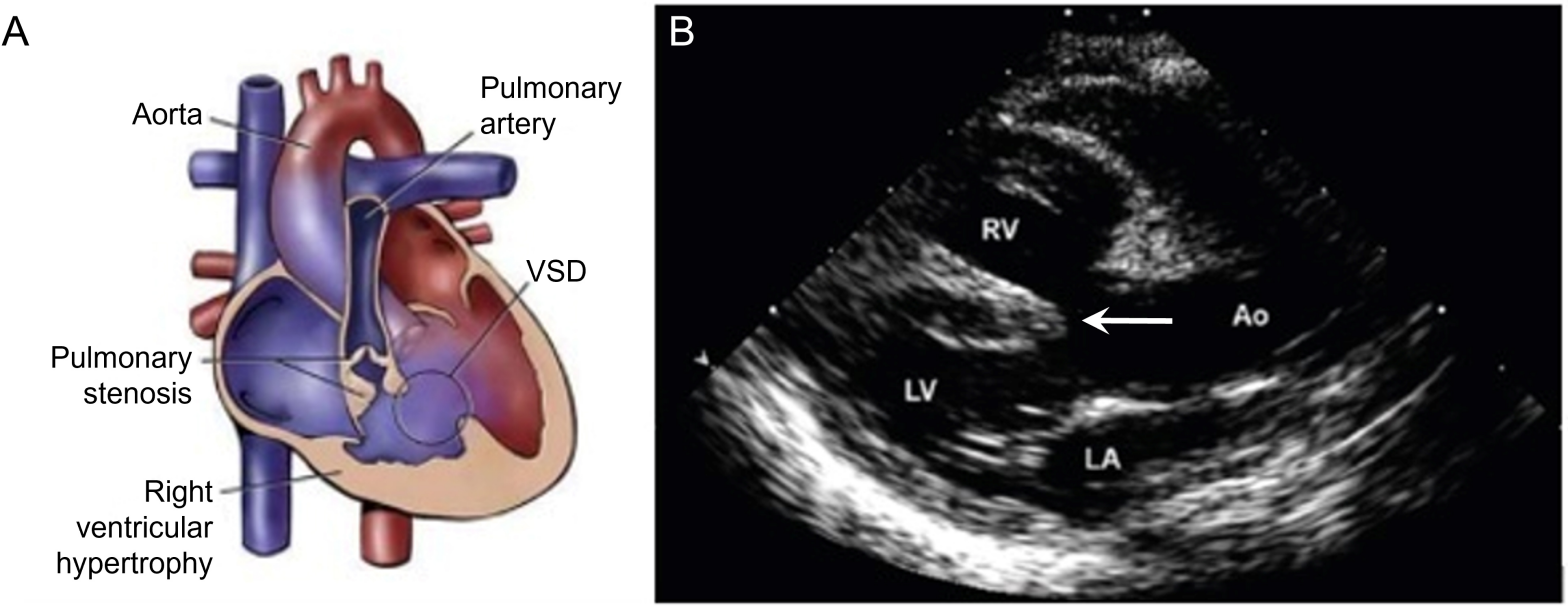
**Tetralogy of Fallot**. (Modified from Vyas HV, Johnson JA, and Eidem BW. Tetralogy of Fallot. In [13]; used with permission of Mayo Foundation for Medical Education and Research.) (A) The 4 anatomic components of tetralogy of Fallot: VSD, right ventricular outflow tract obstruction, overriding aorta, and right ventricular hypertrophy. (B) Parasternal long-axis echocardiographic view showing the VSD (arrow) and overriding aorta. Ao, aorta; LA, left atrium, LV, left ventricle; RV, right ventricle; VSD, ventricular septal defect.

Tetralogy of Fallot (TOF) comprises about 8% to 10% of all CHD, and it is the most common form of cyanotic cardiac disease. TOF is caused by an antero-cephalad deviation of the conal septum, which results in 4 cardiac features: a malaligned VSD; an overriding aorta; RVOTO, usually infundibular with or without PS; and secondary RV hypertrophy (Fig. 5, Ref. [13]).

Patients with TOF have cyanosis because of a right to left shunt through the VSD and concomitant RVOTO; however, the clinical spectrum varies in relation to the extent of the RVOTO. Most patients with TOF undergo at least 1 surgical intervention during childhood, which has evolved from a palliative systemic to PA shunt (Blalock-Thomas-Taussig shunt) to a complete repair, i.e., closing the VSD and relieving the RVOTO, which may involve pulmonary valvotomy, resecting the infundibular stenosis, and placing a transannular outflow patch or, occasionally, a RV to PA conduit if an anomalous coronary artery crosses the RVOT. Common sequelae of complete repair include substantial pulmonary regurgitation, residual RVOTO, residual shunt, RV dysfunction or dilatation, tricuspid valve regurgitation, aortic regurgitation, aortic dilatation, and arrhythmias. Patients with TOF need lifelong follow-up with echocardiography to assess for functional capacity as well as prepregnancy counseling. Cardiac echocardiography can be complemented by CMR or CCT.

**Fig. 6.**
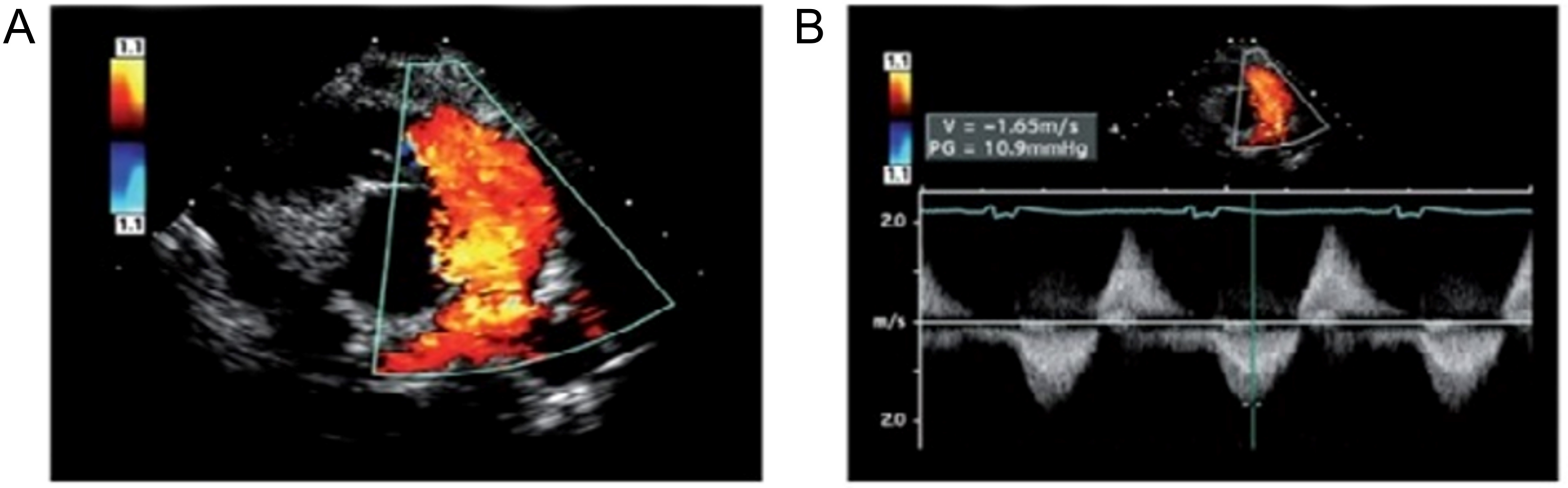
**Echocardiography of severe pulmonary regurgitation in a patient after repair of tetralogy of Fallot**. (From Vyas HV, Johnson JA, and Eidem BW. Tetralogy of Fallot. In [13]; used with permission of Mayo Foundation for Medical Education and Research.) (A) Color Doppler image showing a broad jet of regurgitant flow extending into the branch pulmonary arteries and suggesting severe pulmonary valve regurgitation. (B) Continuous-wave Doppler image showing a dense signal (pulmonary regurgitation) during diastole with a rapid return of the signal to the Doppler baseline, consistent with equalization of the right ventricular and pulmonary arterial pressures. Low V (1.6 m/s) suggests no significant residual right ventricular outflow tract obstruction. PG, peak gradient; V, systolic velocity.

After repair of TOF, the echocardiographic evaluation should include assessment of RV size and function with the latter including fractional area change, tricuspid annular systolic plane excursion, tissue Doppler imaging, myocardial performance index, RV strain, RV diastolic function, and 3D assessment. Assessing the status of the pulmonary valve is also important, and special attention should be paid to the severity of regurgitation, which may be deceiving. Severe pulmonary regurgitation should be suspected when there is low-velocity laminar regurgitant flow, a pulsed-wave Doppler signal of pulmonary regurgitation returning to baseline rapidly (pressure half-time <100 ms suggests clinically significant regurgitation), and diastolic flow reversal in the branch PAs (Fig. 6, Ref. [13]). Moreover, it is important to assess the RV outflow tract for residual stenosis (location of stenosis, peak/mean gradients), the branch PAs for possible distal stenosis, the severity of tricuspid valve regurgitation, the estimated RV systolic pressure, LV size and function, aortic valve regurgitation, dimensions of the ascending aorta, aortic arch sidedness, and residual VSD (typically seen at the margin of the VSD patch by using color flow Doppler; continuous-wave Doppler provides an estimation of the LV to RV pressure gradient) [13].

Pregnancy in patients with unrepaired TOF is rare and not recommended because of poor maternal and fetal outcomes [45]. After a TOF repair, pregnancy tends to be well-tolerated, unless there is substantial RV or LV dysfunction, severe pulmonary regurgitation, severe RVOTO (>2/3 systemic pressure), or severe pulmonary hypertension [15, 46]. Generally, echocardiography should be performed during every trimester to assess the status of postoperative TOF sequelae, accounting for gestational age and the associated physiologic and hemodynamic effects. Common adverse maternal outcomes include arrhythmias and right-sided heart failure, with the risk of these complications being higher for patients with severe pulmonary regurgitation and clinically significant RV dilatation or dysfunction or LV dysfunction [47, 48, 49, 50, 51]. RV enlargement can persist for at least 6 months postpartum, and long-term implications of pregnancy on RV function in patients with TOF are unknown [1, 51].

## 6. Ebstein Anomaly

**Fig. 7.**
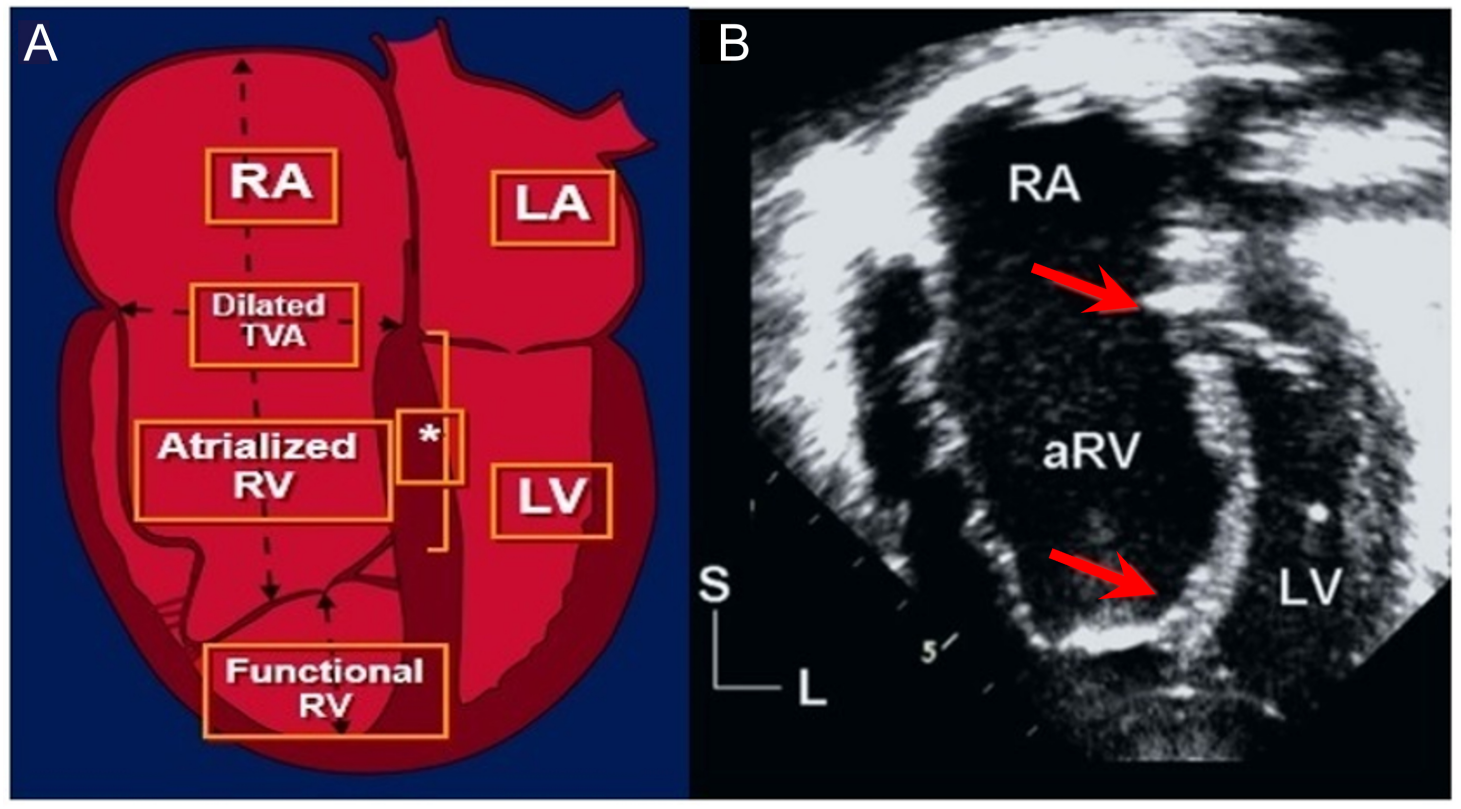
**Ebstein anomaly**. (A, used with permission of Mayo Foundation for Medical Education and Research; B, Modified from O’Leary PW. Ebstein malformation and tricuspid valve diseases. In [13]; used with permission of Mayo Foundation for Medical Education and Research.) (A) Schematic illustration of Ebstein anomaly showing apical displacement of the septal tricuspid valve leaflet (* asterisk represents the displacement index) and tethering of the large (“sail-like”) anterior tricuspid valve leaflet. The right cardiac chambers thus become tripartite with the proper RA, an atrialized portion of the RV, and a functional RV. (B) Systolic 4-chamber apical echocardiographic image delineating measurement of the displacement index between the apically displaced septal tricuspid valve and the mitral leaflet; moreover, it is the distance between the 2 red arrows divided by the patient’s body surface area. An index value >8 mm/$m^{2}$ reliably distinguishes those with Ebstein malformation from patients with other tricuspid abnormalities and from patients with other disorders associated with RV enlargement. aRV, atrialized RV; LA, left atrium; LV, left ventricle; RA, right atrium; RV, right ventricle; TVA, tricuspid valve annulus.

Ebstein anomaly (EA) accounts for <1% of all CHD cases. The anomaly is caused by an abnormality in myocardial development that results in failure of delamination of the tricuspid valve leaflets from the underlying myocardium and thus apical displacement of the annular attachments of the septal and inferior leaflets, variable degrees of tricuspid regurgitation, enlarged right-sided cardiac chambers with a proper right atrium, an atrialized inlet portion of the RV, and a smaller functional RV. The functional annulus of the tricuspid valve is displaced at least 8 mm/$m^{2}$ of BSA—i.e., the distance between the septal tricuspid and anterior mitral leaflet in the apical 4-chamber echocardiographic view (Fig. 7, Ref. [13]); moreover, this displacement is not only a linear downward shift but also a rotational one that follows the RV contour [1, 13, 45, 46, 47, 48, 49, 50, 51]. The anterior tricuspid valve leaflet is abnormal and tends to be large, redundant, fenestrated, and “sail-like”, with variable tethering to the myocardium (Fig. 7A). There is marked dilatation of the tricuspid valve annulus and RV enlargement, with resultant bulging of the ventricular septum leftward and compression of the LV [13, 52]. Most patients with EA have a concomitant interatrial shunt (patent foramen ovale or an ASD) and varying degrees of cyanosis from right to left shunting across the defect caused by elevated right-sided pressures. Other associated cardiac abnormalities include VSD, RVOTO, left-sided heart abnormalities (LV noncompaction, mitral valve prolapse, BAV, abnormal LV morphology), and accessory conduction pathways with increased risk of tachyarrhythmias [52, 53].

Echocardiography is the best method for diagnosing EA. The most sensitive and specific diagnostic feature of EA is the displacement index, which is the distance between the hinge point of the anterior mitral valve leaflet and the septal tricuspid valve leaflet in systole, as seen in the apical 4-chamber view (Fig. 7B). The degree of elongation and tethering of the anterior tricuspid valve leaflet should be assessed along with the location and severity of tricuspid valve regurgitation (color flow, pulsed-wave, and continuous-wave Doppler). The size of the right-sided cardiac chambers and systolic function should also be thoroughly assessed. In addition, echocardiography should be used to evaluate for any associated lesions, such as an interatrial shunt (color flow Doppler imaging with agitated saline contrast), VSD, RVOTO, and LV size and function (systolic and diastolic) [13, 52].

The clinical presentation of patients with EA can vary substantially, based on the extent of apical tricuspid valve leaflet distortion, severity of tricuspid valve regurgitation, the extent of right-sided cardiac chamber dilatation and dysfunction, the presence or absence of a right to left interatrial shunt, and the presence and nature of dysrhythmias [52]. In more severe cases, EA presents at an earlier age. For adults, EA may present with exercise intolerance, dyspnea, cyanosis, right-sided heart failure, arrhythmias, or neurologic events due to paradoxical embolism (transient ischemic attack, stroke, or cerebral abscess). Surgical intervention is indicated for patients with symptoms of heart failure or objective exercise intolerance, severe tricuspid valve regurgitation with progressive RV enlargement or dysfunction, systemic desaturation from a right to left atrial shunt, paradoxical embolism, and/or atrial tachyarrhythmias. In centers with surgical expertise in treating CHD, surgical repair should be considered for patients with EA and may involve tricuspid valve repair if the anatomy is amenable (cone procedure) or tricuspid valve replacement, repair of associated cardiac lesions such as closing an interatrial or ventricular shunt, and relief of RVOTO [15, 46]. An electrophysiologic study can be done preoperatively to determine whether an ablation may be warranted. An isolated interatrial shunt may be closed percutaneously when there are no other indications for surgery.

Patients with EA who want to become pregnant should have pre-pregnancy counseling and cardiac follow-up examinations every trimester and lifelong postpartum. For patients with milder anatomic variants without cyanosis or heart failure, pregnancy is typically well-tolerated [54, 55]; however, patients with severe EA and symptomatic right-sided heart failure or clinically significant cyanosis (oxygen saturation <85%) should be advised against pregnancy because they may be unable to tolerate the increased preload and cardiac output of pregnancy. With increased right atrial pressure, pregnant patients with an interatrial shunt may experience increased cyanosis, a reversal or increase in right to left shunting, and potential for paradoxical embolism [1, 56, 57]. In a study describing EA and pregnancy, no serious maternal cardiac complications were reported [54, 55, 57]. In a literature review, Drenthen *et al*. [47] reported that arrhythmias occurred in 3.9% and heart failure in 3.1% of pregnant patients with EA. However, EA has not been associated with increased risk of premature birth, fetal loss, lower birth weight, or a child with CHD [54, 58, 59].

## 7. Complete (Dextro) Transposition of the Great Arteries

**Fig. 8.**
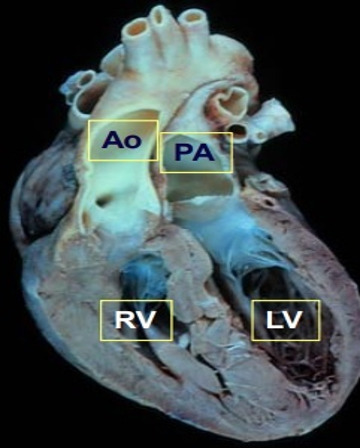
**Transposition (Dextro [D]) of the Great Arteries**. (Used with permission of Mayo Foundation for Medical Education and Research.) In D-transposition of the great arteries, the ventriculoarterial connections are discordant: the aorta arises from the RV, and the PA arises from the LV. Ao, aorta; LV, left ventricle; PA, pulmonary artery; RV, right ventricle.

**Fig. 9.**
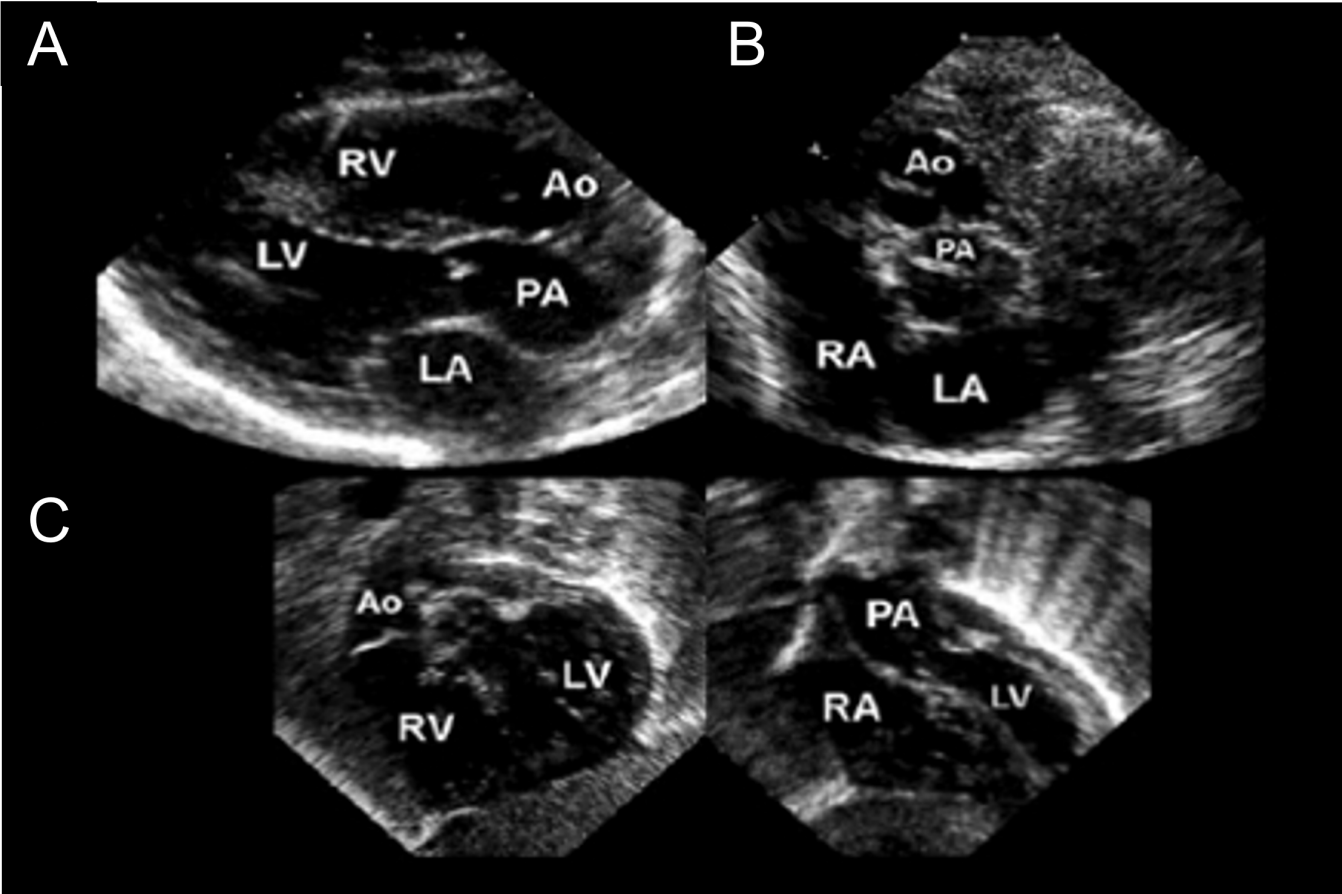
**Echocardiography in Dextro (D)-Transposition of the great arteries**. (Used with permission of Mayo Foundation for Medical Education and Research.) (A) Parasternal long-axis image reveals great vessels parallel with the Ao, arising from the anterior RV and the PA arising from the posterior LV. (B) Parasternal short-axis images showing the Ao anterior and to the right of the PA. (C) Subcostal images show the Ao arising from the RV and the PA arising from the LV. Ao, aorta; LA, left atrium; LV, left ventricle; PA, pulmonary artery; RA, right atrium; RV, right ventricle.

Complete (dextro) transposition of the great arteries (D-TGA) is a common cyanotic cardiac anomaly, comprising about 5% of CHD cases. In D-TGA, the PA arises from the LV, and the aorta arises from the RV (Figs. 8,9). D-TGA is usually associated with a communication between the pulmonary and systemic circulation, e.g., an interatrial shunt, a PDA, or a VSD. Children with D-TGA require surgery to survive into adulthood [52]. The atrial switch procedure (either a Mustard or Senning procedure) was the original standard reparative surgery; however, by the late 1980s, the arterial switch operation became standard of care. When D-TGA includes a VSD and/or LVOTO, the defects may be amenable to other types of surgical repair, such as a Rastelli operation or Kawashima procedure, descriptions of which are beyond the scope of this article.

The atrial switch operation involves creation of and interatrial baffle from the right atrium to the mitral valve and from the left atrium to the tricuspid valve. Hence, the RV continues to serve as the systemic ventricle; oxygenated blood flows from the left atrium through the interatrial baffle and tricuspid valve into the RV and out the aortic valve into the aorta; the systemic venous return is directed to the LV and out the PA. Common long-term sequelae of the atrial switch operation include systemic RV dilation and dysfunction, systemic tricuspid valve regurgitation, baffle obstruction or leak, and arrhythmias [46, 60]. On echocardiography, the great vessels would appear parallel in the parasternal long-axis view with the aorta, which arises from the RV. In the parasternal short-axis view, the aorta is typically anterior and to the right of the PA, which arises from the LV; the PA has fibrous continuity with the mitral valve (Fig. 9) [13]. Adults with D-TGA after an atrial switch procedure should undergo echocardiography at their yearly CHD visits to assess the systemic RV size and function, the systemic tricuspid valve, the systemic and pulmonary venous baffle for stenosis (by color and pulsed-wave Doppler) or leaks (with saline contrast injection), outflow tracts, LV size and function, and vena cavae [13, 52, 60].

The arterial switch operation, the current surgical intervention for repairing D-TGA, entails detaching the aorta and PA and switching their positions, with reimplantation of the coronary arteries. The PA, which arises from the RV, becomes anterior to and straddles the aorta, which arises from the LV. The 20-year survival after an arterial switch procedure is over 90% [61]. Long-term sequelae include dilation of the neoaortic root with possible neoaortic valve regurgitation, branch PA stenosis, supravalvular pulmonary stenosis, coronary artery insufficiency, LV dysfunction, and arrhythmias [46, 62]. Echocardiographic assessment should include assessing biventricular size and function, the neoaortic root, the neoaortic valve, the pulmonary valve and PA, and branch PAs (parasternal and subcostal short-axis imaging plane). CMR or CCT would likely be warranted to supplement echocardiography in assessing the great vessels and coronary arteries during long-term cardiac follow-up and for prepregnancy counseling.

After an arterial switch operation for D-TGA, women desiring pregnancy should undergo a prepregnancy cardiac assessment with a comprehensive echocardiographic examination and should have serial assessments at least every trimester. Pregnancy should be relatively well-tolerated as long as there is no severe impairment of systemic RV function or severe systemic tricuspid valve regurgitation. However, pregnant women have an increased risk of heart failure or arrhythmias. Worsening tricuspid valve regurgitation and an irreversible decline in RV function have also been reported [63, 64, 65, 66, 67]. In a study by Metz *et al*. [68], intracardiac baffle obstruction requiring postpartum stenting occurred in 36% of completed pregnancies.

Data on pregnancy outcomes after an arterial switch procedure for D-TGA are scarce and come from small cohorts of women [69]. However, the risk would seem to be low for women with good functional capacity before pregnancy, preserved ventricular function, no substantial coronary artery compromise, and no clinically significant RVOTO. In a series of 41 pregnant women, heart failure occurred in 1 patient (2.4%) and ventricular tachycardia in 1 (2.4%), and no women died [70]. Closer surveillance, however, would be prudent for women with a dilated neoaorta.

## 8. Congenitally Corrected Transposition of the Great Arteries

**Fig. 10.**
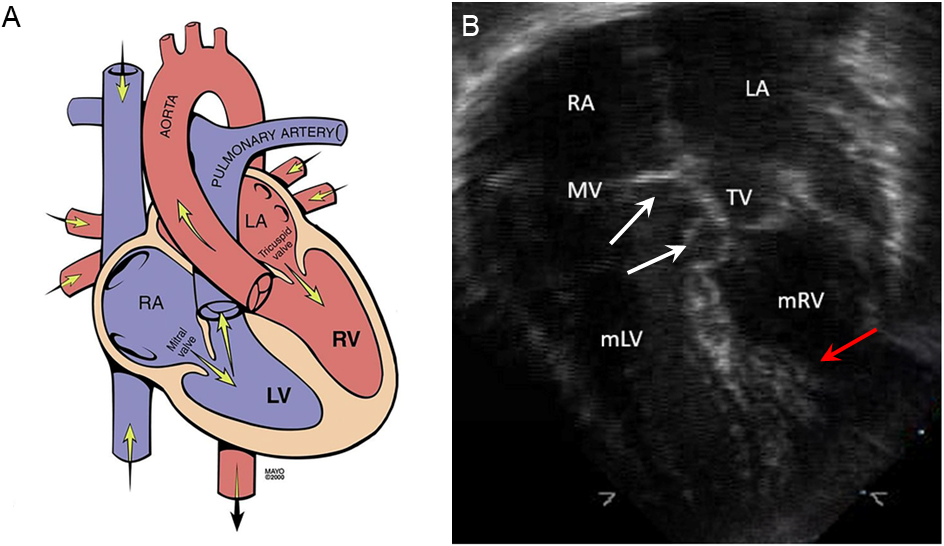
**Congenitally corrected transposition of the great arteries**. (A) Schematic of congenitally corrected transposition of the great arteries showing atrioventricular and ventriculoarterial discordance (Used with permission of Mayo Foundation for Medical Education and Research.) (B) The 4-chamber apical echocardiographic view shows the more apical positioning (white arrows) of the tricuspid valve relative to the mitral valve. The tricuspid valve is somewhat dysplastic. More prominent trabeculations and moderator band (red arrow) help identify the morphologic right ventricle. LA, left atrium; mLV, morphologic left ventricle; mRV, morphologic right ventricle; MV, mitral valve; RA, right atrium; TV, tricuspid valve.

Congenitally corrected transposition of the great arteries (ccTGA) is a rare cardiac anomaly that accounts for 0.4% of CHD cases and involves atrioventricular and ventriculoarterial discordance [56]. It can be thought of as “ventricular inversion”, where systemic venous return empties into the right atrium and traverses the mitral valve to the LV, which ejects blood into the PA. Pulmonary venous return empties into the left atrium and flows through the tricuspid valve into the systemic RV, which ejects blood into the aorta (Fig. 10A). Common associations with ccTGA include an abnormal (Ebstein-like) systemic tricuspid valve, VSD, PS, and abnormal base-apex orientation, especially dextrocardia. Long-term complications are related to progressive subaortic RV dilation and dysfunction, progressive systemic tricuspid valve regurgitation, LVOTO, and acquired heart block.

The clinical presentation depends on the associated malformations. Isolated ccTGA may not present until adulthood, whereas persons with associated malformations likely would have undergone surgical interventions in childhood. In adults, RV dilatation and dysfunction are attributable to systemic tricuspid valve regurgitation, and tricuspid valve replacement is indicated in patients with symptomatic severe tricuspid valve regurgitation with normal or mildly systemic RV dysfunction [15].

Echocardiography is the standard imaging modality for ccTGA and can be supplemented with CCT. A systemic segmental assessment should be followed to delineate systemic venous drainage, atrioventricular connections, ventriculoarterial connections, and the associated malformations. The parasternal windows may be suboptimal for visualizing ccTGA because the great arteries run parallel, and the aorta is anterior and to the left of the PA. Therefore, the subcostal and apical imaging planes are best for visualizing ccTGA. The subcostal window shows the cardiac position and visceral situs. The atrioventricular and ventriculoarterial relationships can be defined in both subcostal and apical planes. Particular attention should be paid to defining the morphologic RV with its unique features, including the apical position of the hinge point of the septal tricuspid valve leaflet relative to the anterior mitral valve leaflet, chordal attachments of the tricuspid valve to the ventricular septum, presence of a moderator band, the trabeculated endocardial surface, and a pyramidal shape to the ventricular cavity (Fig. 10B) [13]. Assessing associated lesions, such as an Ebstein-like tricuspid valve, VSD, and LVOTO, should complete the echocardiographic examination.

As for other patients with complex CHD, women with ccTGA should undergo a complete prepregnancy cardiac evaluation with cardiac imaging and possible stress testing to help stratify their risk with pregnancy. Pregnant patients should be followed up with echocardiography every trimester and more frequently in the latter stages of pregnancy depending on the status of the systemic RV, tricuspid valve, and dysrhythmias. Women with good functional capacity, normal systemic RV function, and no significant tricuspid valve regurgitation are unlikely to experience cardiac complications during pregnancy. Patients should avoid pregnancy if they have severe tricuspid valve regurgitation, systemic ventricular dysfunction (RV ejection function <40%), and poor functional capacity (New York Heart Association classes III and IV) [71]. Worsening systemic RV function (irreversible in 10%), worsening tricuspid valve regurgitation, heart failure, and atrial arrhythmias have been reported for women during pregnancy [67, 71, 72]. Arrhythmias were reported for 3.6% and heart failure for 7.1% of pregnant patients with ccTGA [47].

## 9. Conclusions

Increasing numbers of patients with CHD are surviving to the age of contemplating pregnancy. To better counsel and follow up with these patients, medical practitioners need to be familiar with common CHD lesions and long-term sequelae of the interventions, key echocardiographic and other cardiac imaging findings, and changes during pregnancy. Echocardiography has an important role in assessing CHD for patients before, throughout, and after pregnancy. Fortunately, with proper follow-up, most patients with CHD can have successful pregnancies, albeit with risks to both the mother and baby.

## Abbreviations

ASD, atrial septal defect; AVSD, atrioventricular septal defect; BAV, bicuspid aortic valve; BSA, body surface area; ccTGA, congenitally corrected transposition of the great arteries; CCT, cardiac computed tomography; CHD, congenital heart disease; CMR, cardiac magnetic resonance imaging; 2D, 2-dimensional; 3D, 3-dimensional; D-TGA, complete (dextro) transposition of the great arteries; EA, Ebstein anomaly; LV, left ventricular; LVOT, left ventricular outflow tract; LVOTO, left ventricular outflow tract obstruction; MR, mitral regurgitation; MVSA, membranous ventricular septal aneurysm; PA, pulmonary artery ; PAPVR, partial anomalous pulmonary venous return; PDA, patent ductus arteriosus; PS, pulmonary valve stenosis; Qp:Qs, pulmonary to systemic flow ratio; RV, right ventricular; RVOTO, right ventricular outflow tract obstruction; SVC, superior vena cava; SVR, systemic vascular resistance; TOF, tetralogy of Fallot; TTE, transthoracic echocardiography; VSD, ventricular septal defect.
